# Metabolic syndrome, biochemical markers, and body composition in youth living with perinatal HIV infection on antiretroviral treatment

**DOI:** 10.1371/journal.pone.0230707

**Published:** 2020-03-30

**Authors:** Linda Aurpibul, Sirianong Namwongprom, Tavitiya Sudjaritruk, Sakaewan Ounjaijean

**Affiliations:** 1 Research Institute for Health Sciences, Chiang Mai University, Chiang Mai, Thailand; 2 Department of Radiology, Faculty of Medicine, Chiang Mai University, Chiang Mai, Thailand; 3 Department of Pediatrics, Faculty of Medicine, Chiang Mai University, Chiang Mai, Thailand; 4 Clinical and Molecular Epidemiology of Emerging and Re-emerging Infectious Diseases Cluster, Faculty of Medicine, Chiang Mai University, Chiang Mai, Thailand; China Medical University, TAIWAN

## Abstract

People living with HIV who are on antiretroviral treatment are at increased risk of developing premature cardiovascular disease. Children with perinatal HIV infection (PHIV) have survived through their adolescence and are entering adulthood. We determined the prevalence of metabolic syndrome, abnormal biochemical markers, and characterized body composition parameters in youth living with perinatal HIV infection. This cross-sectional study was conducted at the Research Institute for Health Sciences, Chiang Mai University, Chiang Mai, Thailand from December 2017 to February 2018. PHIV-youths between 15 <25 years of age who were receiving ART were enrolled. Data collection included ART-related history, blood pressure, and anthropometric measurements. Body composition including android, gynoid fat mass, and total body fat were measured by dual-energy X-ray absorptiometry. Fasting blood was drawn to test for lipid profile, glucose, and high sensitivity c-reactive protein (hsCRP). One hundred and twenty PHIV-youths (48% female) were enrolled. Their mean age and the median duration on ART were 20.3 (SD2.6) and 14.1 (IQR 10.4–14.9) years, respectively; 76 (63%) were on first-line non-nucleoside reverse transcriptase inhibitors-based regimens. Thirty-three (28%), 74 (62%), and 13 (11%) of PHIV-youths were underweight (BMI < 18.5 kg/m^2^), normal (BMI 18.5–24.9 kg/m^2^), and overweight (BMI ≥ 25.0 kg/m^2^), respectively. The prevalence of metabolic syndrome was 10.6% (95%CI 5.0–16.0). Seventy-six of 113 (67.3%) of PHIV-youths had lipid alteration; the most prevalent types being low HDL (46.9%) and increased triglycerides (27.4%). Overall 43 (35.9%) had increased hsCRP (16.7% with immediate and 19.2% with high risk for CVD). Females had significantly higher percentage of android and gynoid fat, but lower Android to gynoid ratio (AGR) compared to males. There were 77%, 31%, and 21% of PHIV-youths in the overweight, normal weight, and underweight group with AGR in tertile 3, respectively. In conclusion, we documented presence of metabolic syndrome in 10.6% of PHIV-youths on ART. Increase AGR representing abdominal obesity was detected even in youths with normal BMI or underweight.

## Introduction

Globally, children who acquired HIV infection perinatally have survived through their adolescent years and are now approaching young adulthood [1]. As of 2015, there were approximately 4,500 youths living with perinatal HIV infection (PHIV-youths) on antiretroviral treatment in Thailand [2]. There is a need for long-term health care and surveillance of long-term complications arising either from HIV infection itself or from antiretroviral treatment (ART). Evidence from long-term studies indicate that older PHIV-youths are at risk for premature cardiovascular disease [3].

While data in this population remains scarce, especially in terms of ultimate outcomes, we might extrapolate certain findings from studies in adults living with HIV on ART. There was evidence that adults living with HIV on ART are at increased risk of developing cardiovascular disease associated with unfavorable metabolic profiles [4, 5]. Other studies have also reported associations between metabolic derangement and fat distribution in both adults living with HIV [6] and healthy children [7]. In children with perinatal HIV infection who have been on antiretroviral treatment since childhood, fat redistribution was observed a few years following ART initiation. A previous study conducted by our team identified that 65% of children living with HIV, mean age of 7.6 years, developed lipodystrophy 144 weeks after ART initiation, while dyslipidemia was observed in 11–12% of children [8]. ART is a lifelong commitment and children living with HIV will experience changes in their metabolism and their body over the course of their lifetime. Current clinical practice guidelines recommend monitoring for long-term metabolic abnormalities resulting from ART through biochemical markers in the blood and body mass index (BMI) calculated from body weight and height. It is known that an elevated BMI is a risk factor for metabolic syndrome. Many PHIV-youths have experienced wasting syndrome or growth failure prior to ART initiation [9]. Although PHIV-youths were able to reverse growth deficits after ART initiation, the majority had normal BMI or remained underweight. Thus, they would not ever match the conventional criteria used for identifying metabolic risk and might be misclassified as low risk for metabolic abnormalities. In addition to screening for alterations in lipid and glucose metabolism, screening for cardiovascular disease biomarkers have also been proposed. C-reactive protein is an acute phase reactant that elevates in response to systemic inflammation. It is the most widely evaluated biomarker for global cardiovascular risk prediction [10, 11]. High sensitivity C-reactive protein (hsCRP) might also be useful in setting that the test is accessible.

Truncal or abdominal adiposity, which reflects android body type, is also a known predictor of metabolic syndrome and cardiovascular disease. It can be assessed clinically by anthropometric measurements including waist circumference, waist-to-hip, or waist-to-height ratio. In addition, imaging studies i.e. computerized tomography, magnetic resonance imaging, and dual-energy X-ray absorptiometry (DXA) could also be used. The latter may be the most feasible option as it is the least expensive, can be performed quickly, and exposes patients to a minimal amount of ionizing radiation. DXA android percent fat with waist circumference was reported as a good predictor for visceral fat measured by MRI in a Japanese study among pre-adolescent girls [12]. Increased abdominal fat may indicate higher metabolic risk.

The objectives of this study were to determine the prevalence of metabolic syndrome and abnormal biochemical markers, as well as to characterize body composition parameters in PHIV-youths classified as having a low, normal, or high body mass index. We also explored the correlation between anthropometric measurements, biochemical markers for cardiovascular disease, and body composition parameters in this population.

## Materials and methods

### Study design and population

The cross-sectional study was conducted at the Research Institute for Health Sciences, Chiang Mai University, Chiang Mai, Thailand from December 2017 to February 2018. Inclusion criteria were: 1) aged between 15–25 years, 2) having documented perinatal HIV infection or diagnosed with HIV infection prior to being sexually active, and 3) receiving ART at the time of study enrollment. Those with acute illnesses or chronic conditions that prohibited him/her from performing study activities in the clinic were excluded. Study participants were recruited from the pediatric HIV clinic at the university hospital affiliated with Chiang Mai University and from HIV clinics in other neighboring hospitals. Participants were approached during their routine visit for HIV care.

### Data collection, tools, and measurements

Data collection included ART-related history from medical records, blood pressure, and anthropometric measurements performed by trained study staff. Blood pressure was measured in a sitting position following a 5-minute rest using an automated machine. Repeat measurements were made for high readings after a 5-minute interval. Weight and height were measured while participants wore lightweight clothing and no shoes using standard instruments. BMI was calculated by dividing body weight (in kilograms) by height (in meters) squared (BMI = weight/height^2^). Underweight and overweight were defined as having BMI < 18.5 and > 25 kg/m^2^, respectively [13]. Waist circumference was measured at the approximate midpoint between the lower margin of the last palpable rib and the top of the iliac crest (or one centimeter above umbilical level in participants whose bony landmark was not palpable) in standing position as the participant breathed out normally. The study followed the International Diabetes Federation cut-off points for South Asians, Chinese and Japanese, with waist cut-offs of > 80 for females and > 90 centimeters for males [14]. Hip circumference was measured around the widest portion of the buttocks. Waist-to-hip ratio (WHR) and waist-to-height ratio (WHtR) were calculated for each participant.

**Fig 1:**
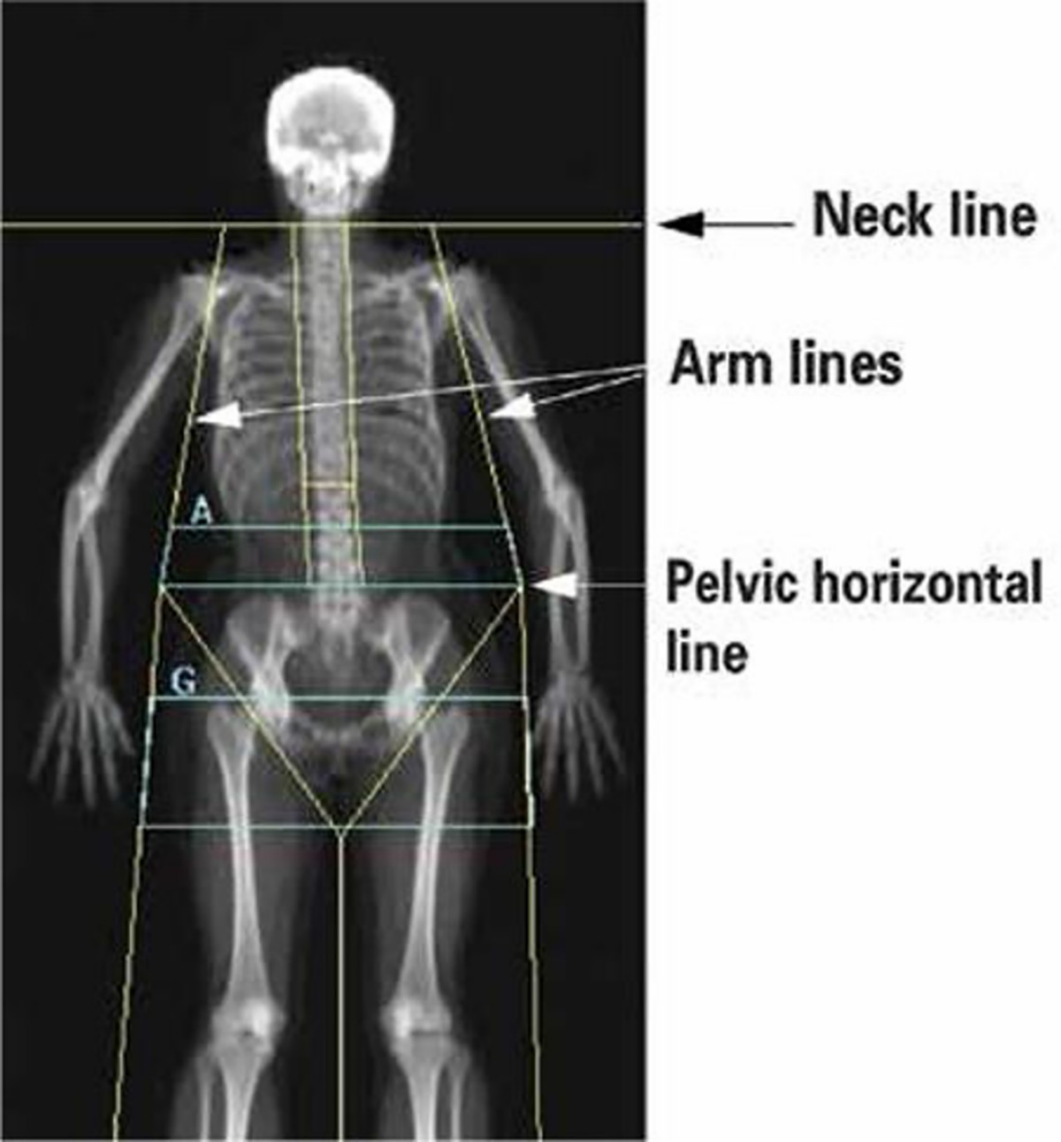
Andriod and gynoid regions of interest (ROI).

Body composition including android (AFM), gynoid (GFM) fat mass, and total body fat were measured by a dual-energy X-ray absorptiometry (DXA) machine (Hologic Discovery A, Hologic Inc., Badford, MA) equipped with software version 12.3. The machine was calibrated daily using a standard phantom provided by the manufacturer. The *in vivo* precision of the machine was 2.0%. Regional body composition of interest was defined using the manufacturer’s software. Android height is 20% of distance from pelvic horizontal cut line to neck cut line using the arm cut lines as lateral boundaries. Gynoid height is twice the height of the android region using leg cut lines as lateral boundaries (Fig 1), with the upper boundary below the pelvic horizontal cut line by 1.5 x the height of the android region height. The android to gynoid ratio (AGR) was computed using fat percentage in the A and G regions divided into tertiles to demonstrate trends.

Blood specimens were obtained after 12-hour fasting. Lipid profile (triglyceride (TG), total cholesterol, high density lipoprotein cholesterol (HDL), and low density lipoprotein cholesterol (LDL)), and glucose were performed at the Faculty of Associated Medical Sciences, Chiang Mai University;hsCRP level was measured at the nutrition analysis laboratory at the Research Institute for Health Sciences, Chiang Mai University. The test kit used was C-Reactive Protein ELISA Kit, Human, CYT298 (Merck Millipore) which detects CRP levels as low as 0.1 mg/L.

Metabolic syndrome was defined using criteria set by the National Cholesterol Education Program–Adult Treatment Panel III report (NCEP-ATP III) (Table 1) [15]. We used waist-to-height ratio in this study as some participants have not yet reached their final height. Diagnosis of metabolic syndrome was made if three of the five criteria were met. Lipid alteration was defined as having TG ≥ 150 mg/dL, total cholesterol > 200 mg/dL, LDL ≥ 130 mg/dL or HDL < 40 mg/dL in males or < 50 mg/dL in females. Abnormal glucose was defined as glucose > 100 mg/dL. For hs-CRP, the cut-off > 1 mg/L was defined as having risk for cardiovascular disease according to the American Heart Association/Centers for Disease Control working Group on markers of inflammation in CVD [16].

**Table 1:** Criteria for clinical diagnosis of metabolic syndrome based on ATP III.

Measurements (any 3 of 5 constitute diagnosis of metabolic syndrome)	Categorical cut-off points
Elevated waist circumference (Asians)*	≥ 90 cm in male, ≥ 80 cm in female
Elevated triglycerides	≥ 150 mg/dL, or on drug treatment for elevated triglycerides
Reduced HDL cholesterol	< 40 mg/dL in male, or < 50 mg/dL in female, or on drug treatment for reduced HDL cholesterol
Elevated blood pressure	≥ 130 mmHg systolic blood pressure and/or ^3^ 85 mm Hg diastolic blood pressure or on antihypertensive drug treatment
Elevated fasting glucose	≥ 100 mg/dL or on drug treatment for elevated glucose

* alternative variable is waist-to-height ratio ≥ 0.5 either in male or female.

### Ethical considerations

The study was approved by the institutional review boards at the Research Institute for Health Sciences, Chiang Mai University (Certificate approval number 48/2017), as well as at the Chiang Mai University Faculty of Medicine. Written informed consent was obtained from each participant prior to enrollment. Parental consent and assent were obtained for those under 18 years of age.

### Statistical analysis

Descriptive statistical analysis was performed using SPSS Statistics for Windows, Version 17.0. Chicago: SPSS Inc; 2008. Continuous variables were presented as mean (SD) or median (range or interquartile range, IQR), as appropriate. Categorical variables were presented in number (percentage). Student’s t-test was used to compare continuous variables while either chi-square or Fisher’s exact test were used to compare categorized variables. The five metabolic risk components were scored 0 (no presence of risk) or 1 (presence of risk) using ATPIII criteria. Sum of scores was used in analysis. Linear regression was applied to determine relationship between hsCRP, android and gynoid fat mass, and AGR with metabolic risk components scores adjusted for age, sex, and BMI. A p-value of <0.05 for two-sided tests was considered to be statistically significant.

## Results

### Characteristics of study participants

One hundred and twenty PHIV-youths were enrolled; 58 (48%) were female. Table 2 provides participant demographic information. The mean age of the participants was 20.3 ± 2.6 years. The median duration of time on ART was 14.1 (interquartile range, IQR 10.4–14.9) years. Seventy-six (63%) were on first-line non-nucleoside reverse transcriptase inhibitors (NNRTI), either nevirapine- or efavirenz-based regimen. The overall self-reported adherence ranged from 70–100%; males reported significantly lower adherence compared to females (80% (IQR 80–100) vs. 90% (IQR 58–100), p = 0.04). All had arterial blood pressure within normal range, while two males were on antihypertensive drugs at the time of the study. Thirty-three (28%), 74 (62%), and 13 (11%) of PHIV-youths in this study were underweight (BMI < 18.5 kg/m^2^), normal (BMI 18.5–24.9 kg/m^2^), or overweight (BMI ≥ 25.0 kg/m^2^), respectively. Almost all (12 of 13, 92%) overweight youths were male, while 24 of 33 (73%) underweight youths were female. All participants underwent DXA, though seven participants did not have blood tests for biochemical markers for this study.

**Table 2:** Demographic data of study participants stratified by sex.

Variables	Total	Female	Male	p-value
Number of participants	120	58 (48)	62 (52)	
Age (years), mean (SD)	20.3 (2.6)	20.3 (2.6)	20.2 (2.6)	0.92
15-<18 years	28 (23)	14 (24)	14 (23)	0.84
18–25 years	92 (77)	44 (76)	48 (77)	
Duration on ART, years	14.1 (10.4–14.9)	14.4 (10.6–14.9)	13.8 (9.7–14.8)	0.17
Duration on ART, months	169 (125–179)	172 (127–179)	165 (116–178)	0.17
Current ART regimens				
nevirapine-based	22 (18)	6 (10)	16 (26)	0.04
efavirenz-based	54 (45)	25 (43)	29 (47)	
PI-based regimens	38 (32)	22 (38)	16 (26)	
other regimens	6 (5)	5 (9)	1 (2)	
Self-reported adherence to ART (%)	90 (70–100)	90 (80–100)	80 (58–100)	0.04
Systolic blood pressure	117 (108–125)	110 (101–118)	121 (114–128)	<0.01
Diastolic blood pressure	70 (64–75)	68 (63–72)	71 (66–79)	<0.01
Body mass index (kg/m^2^)				
< 18.5 (underweight)	33 (28)	24 (41)	9 (15)	<0.01
18.5–24.9 (normal)	74 (62)	33 (57)	41 (66)	
> 25.0 (overweight)	13 (11)	1 (2)	12 (19)	

Data in mean ± standard deviation, median (interquartile range), or number (%) as appropriate

P-value from t-test, chi square, or fisher exact as appropriate

IQR interquartile range; ART antiretroviral treatment; PI protease inhibitor; NNRTI non-nucleoside reverse transcriptase inhibitors

### Prevalence of metabolic syndrome and abnormal biochemical markers

The prevalence of metabolic syndrome was 10.6% (95% confidence interval CI 5.0–16.0) using the waist-to-height ratio criterion. There were seven participants who did not have blood drawn for lipid profile. Seventy-six of 113 (67.3%) of PHIV-youths had lipid profile alteration (Table 3) with the most prevalent type being low HDL; 65% in females, and 31% in males. Low HDL was evidenced in 46.9% of participants and a higher proportion of PHIV-youths who were underweight had low HDL (56.7%) than those who had a normal BMI (47.1%) or were overweight (23.1%), although these differences were not statistically significant (p = 0.128). Only 27.4% of participants had high triglycerides, though overweight participants had worse outcomes with higher rates of high triglycerides (61.5%), and high LDL (46.2%). Differences in triglycerides and LDL were significantly higher in the overweight group than in participants with normal BMI or underweight. There was no difference in mean glucose level between the three groups and only 2 participants had high glucose (111 mg/dL in one and 131 mg/dL in another), both with normal BMI. No participant was taking medication for elevated glucose, while one male participant was on anti-lipid agents, and two males were on anti-hypertensive drugs.

**Table 3:** Metabolic syndrome, lipid profile and high-sensitivity c-reactive protein in participants with low, normal, and high body mass index.

Variables	Total	Body mass index (kg/m^2^)			p-value
		< 18.5	18.5–24.9	> 25.0	
**Number of participants**	**113**	**30**	**70**	**13**	** **
Prevalence of metabolic syndrome	12 (10.6)	0	6 (8.6)	6 (46.2)	< 0.001
Prevalence of lipid profile alteration	76 (67.3)	21 (70.0)	44 (62.9)	11 (84.6)	0.319
**Biochemical parameters**					
Triglyceride (mg/dL)	133 (102)	106 (44)	141 (122)	154 (69)	0.212
Triglyceride > 150 mg/dL	31 (27.4)	4 (13.3)	19 (27.1)	8 (61.5)	0.007
Total cholesterol (mg/dL)	171 (37)	154 (35)	172 (35)	204 (27)	< 0.001
Total cholesterol > 200 mg/dL	22 (19.5)	2 (6.7)	12 (17.1)	8 (61.5)	< 0.001
HDL-cholesterol (mg/dL)	45 (12)	46 (11)	45 (12)	46 (12)	0.946
HDL-cholesterol < 40 mg/dL (male)	19/61 (31)	3/8 (38)	13/41 (32)	3/12 (25)	0.844
HDL-cholesterol < 50 mg/dL (female)	34/52 (65)	14/22 (64)	20/29 (69)	0	0.424
LDL-cholesterol (mg/dL)	100 (31)	87 (32)	101 (28)	128 (29)	< 0.001
LDL-cholesterol > 130 mg/dL	15 (13.3)	3 (10.0)	6 (8.6)	6 (46.2)	<0.001
Glucose (mg/dL)	84 (8)	82 (6)	85 (9)	84 (6)	0.186
Glucose > 100 mg/dL	2 (1.8)	0	2 (2.9)	0	1.000
High sensitivity C-reactive protein, hsCRP (mg/dL)	2.28 (3.79)	2.10 (3.99)	1.84 (3.34)	5.07 (4.68)	0.017
hsCRP 1-< 3 mg/dL (intermediate risk for CVD)	20 (16.7)	2 (6.7)	12 (17.1)	6 (46.2)	0.002
hsCRP ≥ 3 mg/dL (high risk for CVD)	23 (19.2)	7 (23.3)	11 (15.7)	5 (38.5)	
**DXA**					
Percent android fat (%)	24.5 (8.10)	22.66 (5.77)	24.02 (8.60)	32.04 (6.20)	< 0.01
Percent gynoid fat (%)	27.21 (8.17)	27.92 (7.11)	26.81 (9.09)	27.71 (4.71)	0.79
Total body fat (%)	23.41 (7.05)	22.65 (4.90)	23.11 (8.05)	27.08 (4.29)	0.13
Android/gynoid ratio (AGR)	0.91 (0.19)	0.83 (0.14)	0.90 (0.14)	1.18 (0.29)	< 0.01
AGR tertile 1		18 (55)	20 (27)	2 (15)	< 0.01
AGR tertile 2		8 (24)	31 (42)	1 (8)	
AGR tertile 3		7 (21)	23 (31)	10 (77)	

Data in mean (standard deviation), or number (%)

p-value by student t-test, Chi square, or fisher exact as appropriate

There were 7 participants who did not have blood tests (3 in underweight and 4 in normal BMI groups)

Overall 43 (35.9%) had high levels of hsCRP (16.7% with immediate and 19.2% with high risk for CVD). The percentage of PHIV–youths with high risk for CVD were 23.3%, 15.7%, and 38.5% in the underweight, normal BMI, and overweight groups, respectively. The 12 PHIV-youths with metabolic syndrome ranged in age from 16.6 to 24.8 years, and their duration on ART ranged from 6.6 to 15.6 years. Eleven (92%) were male, six (50%) were overweight and all but one, were on NNRTI-based ART. Ten of the 12 participants with metabolic syndrome had high levels of hsCRP (83%).

### Anthropometric measurement and body composition parameters

Overall 15 (13%) youths had a high waist circumference and 26 (22%) were above the WHR cut-off. Three (9%) youths who were underweight had increase WHR, while none had increase WC or WHtR. Of the participants with normal BMI, 18% and 19% had increase WHR or WHtR, respectively (Table 4).

**Table 4:** Anthropometric measurement and body composition parameters of youths living with perinatal HIV infection stratified by sex.

Variables	Total	Female	Male	p-value^d^
Number of participants	120	33 (28)	74 (62)	
Waist circumference, WC (cm)	73.01 (11.01)	69.44 (7.03)	76.35 (12.91)	< 0.001
Increase WC^a^	15 (13)	6 (10)	9 (15)	0.49
Waist-to-hip ratio, WHR	0.82 (0.07)	0.80 (0.06)	0.84 (0.07)	0.007
Increase WHR^b^	26 (22)	14 (24)	12 (19)	0.525
Waist-to-height ratio	0.45 (0.06)	0.45 (0.05)	0.46 (0.07)	0.365
Increase WHtR ratio^c^	26 (22)	12 (21)	14 (23)	0.802
Total body fat (%)	23.41 (7.05)	28.05 (5.46)	19.07 (5.43)	< 0.001
Percent android fat (%)	24.50 (8.10)	28.36 (6.66)	20.93 (7.70)	< 0.001
Percent gynoid fat (%)	27.21 (8.17)	33.57 (5.16)	21.26 (5.56)	< 0.001
Android/gynoid ratio (AGR)	0.91 (0.19)	0.84 (0.14)	0.98 (0.20)	< 0.001

Data in mean ± standard deviation, or number (%) as appropriate

DXA dual energy x-ray absorptiometry; kg kilograms

^a^ ≥ 80 cm on females or ≥ 90 cm in males

^b^ ≥ 0.9 in males, ≥ 0.85 in females

^c^ ≥ 0.5 in both males and females

^d^ P-value from t-test, chi square, or fisher exact as appropriate

The body composition and regional fat distribution parameters are shown in Tables 3 and 4. Overweight PHIV-youths had a significantly higher percentage of android fat than the other two groups, though there was no significant difference in the percentage of total body fat. Female youths had significantly higher percent android and gynoid fat, but lower AGR when compared to male youths. There were 77%, 31%, and 21% of PHIV-youths in the overweight, normal weight, and underweight group with AGR in tertile 3, respectively.

### Correlation between biochemical markers, anthropometric measurements, and body composition parameters

Simple linear regression showed a significant positive correlation between AGR and triglycerides (R^2^ = 0.100), total cholesterol (R^2^ = 0.049), and hsCRP (R^2^ = 0.267) at p-value < 0.001. Total cholesterol also had a significant positive correlation with percentage of android fat. Percent of total body and android fat, as well as AGR were positively correlated with anthropometric measurements (BMI, WC, WHR, WHtR) as shown in Table 5. The univariate analysis revealed significant positive associations with metabolic risk components’ score was found for AGR (β = 2.628), hsCRP (β = 0.080), AFM (β = 0.001) and GFM (β = 0.000). However, after adjusting for confounding variables, the positive correlations did not exist for GFM and hsCRP (Table 6).

**Table 5:** Correlation between biochemical markers, anthropometric measurements, and body composition parameters.

Biomarkers and anthropometric parameters	Percentage of total body fat	Percentage of android fat	Android/gynoid ratio
***Biomarkers***			
** Triglyceride**	0.000 (0.950)	0.019 (0.145)	0.100 (0.001)
** Cholesterol**	0.017 (0.166)	0.045 (0.024)	0.049 (0.018)
** HDL-cholesterol**	0.033 (0.056)	0.016 (0.177)	0.010 (0.286)
** LDL-cholesterol**	0.009 (0.321)	0.023 (0.105)	0.025 (0.096)
** hsCRP**	0.021 (0.123)	0.033 (0.056)	0.267 (0.004)
***Anthropometric measurement***			
** Body mass index**	0.099 (< 0.001)	0.229 (< 0.001)	0.359 (< 0.001)
** waist circumference**	0.080 (0.001)	0.224 (< 0.001)	0.415 (< 0.001)
** waist-to-hip ratio**	0.046 (0.011)	0.166 (< 0.001)	0.379 (< 0.001)
** waist-to-height ratio**	0.218 (< 0.001)	0.389 (< 0.001)	0.347 (< 0.001)

Data are Coefficient of Determination (R-squared) and P-value from simple linear regression

**Table 6:** Univariable and multivariable linear regression analysis of high sensitivity C-reactive protein, android fat mass, gynoid fat mass, and android to gynoid ratio against the metabolic syndrome risk components.

Parameters		Metabolic syndrome risk components	
		Univariable	Multivariable*
**hsCRP**	β	0.080	0.041
	95% CI	0.032–0.129	-0.006–0.089
	p-value	0.001	0.089
**AFM**	β	0.001	0.001
	95% CI	0.001–0.001	0.000–0.001
	p-value	< 0.001	0.023
**GFM**	β	0.000	0.000
	95% CI	0.000–0.001	0.000–0.000
	p-value	0.001	0.167
**AGR**	β	2.628	1.904
	95% CI	1.721–3.535	0.801–3.006
** **	p-value	< 0.001	0.001

hsCPR high sensitivity C-reactive protein; AFM android fat mass; GFM gynoid fat mass; AGR android to gynoid ratio

*adjusted for age, sex, and body mass index

## Discussion

We documented the presence of metabolic syndrome in PHIV-youths who had been taking ART since their childhood, with nearly two-thirds on first line NNRTI-based regimens. Abdominal obesity was also detected not only in overweight PHIV-youths, but also in those with normal BMI or who were underweight. A significant positive correlation between AGR and triglyceride, total cholesterol, and hsCRP was seen, as well as a significant positive association with metabolic risk components’ score was found for AGR and AFM.

In this study, the prevalence of metabolic syndrome in PHIV-youths was 10.6%. This was in line with a previous Thai study that reported a 10% prevalence of metabolic syndrome [17]. That study also had a similar number of females and males, however their participants were younger than those in our study, (median age of 16.7 vs. 20.3 years, respectively), had a shorter median duration on ART (114.3 vs. 169 months, respectively), and all participants were receiving PI-based ART as opposed to 32% in our study. The prevalence of metabolic syndrome in a French national cohort of young adults with perinatal HIV infection was 13.2% in males and 10.4% in females [18]. The population in their study was slightly older than ours with a median age of 23 years, and various ART regimens were used.

The prevalence of lipid alteration in this study was 67.5%, which is similar to the 70% found in a previous Thai study. The differences in the type of lipid change experienced may be due to the different ART regimens used. The majority of our study PHIV-youths (63%) were on NNRTI-based regimen and the prevalence of high triglyceride was only 27.4%; the most prevalent type of lipid alteration was low HDL, especially in females, in which the cut-off was higher than in males. This differs from a previous Thai study which was conducted in a cohort of youths on PI-based regimens. This group experienced hypertriglyceridemia as the most prevalent type of abnormal lipid metabolism (43.8%) and 42.5% and 8.8% had abnormal glucose metabolism and high blood pressure, respectively. In our study we only screened for pre-diabetes using fasting glucose; however, we found only one participant with high glucose in the diabetic range. No participants had high blood pressure at the time of the study, though 2 males (1.6%) were on antihypertensive agents.

Presently, NNRTI-based regimens are the first line ART in children, adolescents, and adults in most resource limited countries including Thailand due to its affordable cost and wide availability. NNRTI-based regimens have a lower risk of metabolic and cardiovascular disease when compared to PI-based regimens [19]. However, our results showed metabolic change in older PHIV-youths receiving NNRTI-based regimen long-term. In addition to risk for atherosclerosis and cardiovascular disease, metabolic syndrome has been a strong predictor of cognitive impairment and a risk factor for HIV-associated neurocognitive disorders [20]. Many comorbidities in people living with HIV who were otherwise stable on ART are increasingly explained through systemic inflammation. Studies in adults and children living with HIV have documented an association between metabolic derangements and systemic inflammatory markers [21, 22]. Inflammation was reported as associated with risk of depression and cancers [23, 24]. In order to early diagnose the long-term effects of ART, screening for organ system involvement is as important as monitoring metabolic changes.

We documented that 35.9% of PHIV-youths in our study had high hsCRP, which inferred risk of CVD. This corroborates a Brazilian study which reported a significantly higher CRP than healthy control among PHIV-children, mean age 12.2 years [25]. However, a Spanish study in PHIV-children with a mean age of 14.2 years and a mean duration of 3 years on ART, reported that the mean hsCRP level in those without metabolic disturbances was similar to non HIV controls [26]. We believe the differences found in our study are due to the older age of our participants and the fact that they have been on ART for a longer period of time. A direct comparison to other pediatric studies conducted in developed countries might not be possible due to large age difference. It might more appropriate if our findings are considered against studies involving adults as guidance for long-term follow-up. HsCRP is a known systemic inflammatory marker that plays a role in the pathogenesis of atherosclerosis and levels of > 1 mg/dL indicate risk for CVD. Studies in adults documented an association between pro-inflammatory biomarkers and the presence of hypertension, metabolic syndrome, coronary artery disease, peripheral artery disease, as well as cerebrovascular events [27]. Although those ultimate outcomes were not expected in a younger population, we observed high hsCRP in 10 out of 12 (83%) PHIV-youths with metabolic syndrome. Given that we found 30% of underweight and 32.8% of normal BMI PHIV-youths also had high hsCRP, and apart from triglyceride and cholesterol, hsCRP was significantly positively correlated with AGR in this study. High levels of hsCRP means that the inflammatory process presents and also implies risk for CVD. In settings where laboratory tests are available, periodical monitoring of hsCRP might help to identify youths with increased risk for metabolic / cardiovascular complications.

One Thai study involving young adults without HIV, mean age 20.4 years, revealed that AGR was a stronger predictor of metabolic syndrome than waist circumference or android fat mass [28]. This association between android fat deposits and metabolic syndrome was previously established in older adults [29]. In this study we found that 77% of PHIV-youths in the overweight group had AGR in tertile 3, which was not surprising. However, 31% of normal BMI and 21% of underweight youths also had AGR in tertile 3. In other words, 57.5% PHIV-youths with AGR in tertile 3 had normal BMI. According to an Indian study among healthy children and adolescents, mean age 11.2 years, 8% of those with normal BMI had abnormal biochemical parameters (either glucose or lipid profiles) [30]. This might be due to fat redistribution in HIV-infected individuals on ART, and its significance as a predictor of metabolic risk required further study. With higher percentage of gynoid fat, female had lower AGR when compared to male PHIV-youths. In this study, almost all participants with metabolic syndrome were male. Lifestyle modification and healthy behaviors such as smoking cessation, exercise, and eating health foods are recommended to reduce other modifiable metabolic risk. This advice is appropriate for both males and females since the study by Sharma found metabolic changes in female youth. The study evaluated changes in body composition measured by dual x-ray absorptiometry (DXA) in a cohort of 156 PHIV youth and 79 HIV negative controls over a 7-year period [31]. They reported that apart from the gains in weight and body mass index, PHIV females demonstrated an unfavorable change in fat redistribution and percent body fat over time that exceeded what was found in PHIV males and females without HIV, which was statistically significant.

A study comparing total body fat and its distribution in PHIV and HIV-exposed uninfected children between 7–16 years of age found that despite of significantly lower BMI and total body fat measured by anthropometry and dual-energy X-ray absorptiometry, the body fat distribution in PHIV-children followed the pattern associated with cardiovascular disease risk [32]. Although the prevalence of hypertension in this study was low, it might increase with age as these children approach adulthood. A Portuguese study reported significantly higher total fat, central, and central/peripheral fat mass ratio in HIV-infected individuals with hypertension than those with normal blood pressure [33]. Although the prevalence of hypertension was greater in HIV-infected patients with lipodystrophy, it was significantly associated with central fat ratio, or AGR in our case. A Brazilian study claimed that patients with normal metabolic profile and clinically diagnosed HIV-associated lipodystrophy syndrome had high total body fat percentages and areas of visceral fat [34]. Thus, HIV infected individuals who do not have abnormal biochemical markers remained at risk for development of cardiovascular disease when assessed using other measures.

This study had several limitations. First the study used a cross-sectional design and we did not have baseline data prior to ART for comparison making it impossible to infer the causality of the effect of HIV infection and ART on the findings. Second, we had no control group. Certain findings might be normal for certain age groups in an ever changing environment. Last, we did not perform lab tests commonly used in screening for pre-diabetes (i.e. insulin level, oral glucose tolerance test). As some findings might be transient and vary, a follow-up study would be useful in gathering more information about metabolic change in this population. Future study to see the association between socioeconomic factors, lifestyle, health behaviors including diet intake and metabolic syndrome/lipid alteration might be warranted in order to design a proper intervention and/or recommendations for this population.

We documented the presence of metabolic syndrome in PHIV-youths who were initiated ART during childhood. Increased AGR indicated abdominal obesity was detected not only in overweight PHIV-youths, but also in those with normal BMI or who were underweight. Apart from HIV treatment outcome monitoring, we should also continue screening PHIV-youths for metabolic and cardiovascular risk factors in addition to screening for neurocognitive and mental health outcomes.

## Supporting information

S1 Dataset (SAV)
